# Mst1-Deficiency Induces Hyperactivation of Monocyte-Derived Dendritic Cells via Akt1/c-myc Pathway

**DOI:** 10.3389/fimmu.2019.02142

**Published:** 2019-09-11

**Authors:** Kyung-Min Cho, Myun Soo Kim, Hak-Jun Jung, Eui-Ju Choi, Tae Sung Kim

**Affiliations:** ^1^Department of Life Sciences, College of Life Sciences and Biotechnology, Korea University, Seoul, South Korea; ^2^Institute of Convergence Science, Korea University, Seoul, South Korea

**Keywords:** Mst1, dendritic cells, hyperactivation, Akt1, c-myc, GM-CSF

## Abstract

Mst1 is a multifunctional serine/threonine kinase that is highly expressed in several immune organs. The role of Mst1 in the activation of dendritic cells (DCs), a key player of adaptive immunity, is poorly understood. In this study, we investigated the role of Mst1 in GM-CSF-induced bone marrow-derived DCs and the underlying mechanisms. *Mst1*^−/−^ DCs in response to GM-CSF expressed higher levels of activation/maturation-related cell surface molecules, such as B7 and MHC class II than *Mst1*^+/+^ DCs. Furthermore, the expression of proinflammatory cytokines, such as IL-23, TNF-α, and IL-12p40, was increased in *Mst1*^−/−^ DCs, indicating that Mst1-deficiency may induce the hyperactivation of DCs. Additionally, *Mst1*^−/−^ DCs exhibited a stronger capacity to activate allogeneic T cells than *Mst1*^+/+^ DCs. Silencing of Mst1 in DCs promoted their hyperactivation, similar to the phenotypes of *Mst1*^−/−^ DCs. *Mst1*^−/−^ DCs exhibited an increase in Akt1 phosphorylation and c-myc protein levels. In addition, treatment with an Akt1 inhibitor downregulated the protein level of c-myc increased in Mst1-deficient DCs, indicating that Akt1 acts as an upstream inducer of the *de novo* synthesis of c-myc. Finally, Akt1 and c-myc inhibitors downregulated the increased expression of IL-23p19 observed in Mst1-knockdown DCs. Taken together, these data demonstrate that Mst1 negatively regulates the hyperactivation of DCs through downregulation of the Akt1/c-myc axis in response to GM-CSF, and suggest that Mst1 is one of the endogenous factors that determine the activation status of GM-CSF-stimulated inflammatory DCs.

## Introduction

Dendritic cells (DCs) orchestrate immune responses, linking innate to adaptive immunity. DCs, which are important professional antigen-presenting cells (APCs) depending on their maturation state, take up a broad range of antigens and present them to T cells. Several endogenous and exogenous stimuli, such as Toll-like receptor (TLR) ligands and inflammatory cytokines, phenotypically and functionally activate DCs. Upon activation, DCs display phenotypic changes, including upregulation of the expression of MHC II and costimulatory molecules, and changes in expression patterns of chemotactic and homing receptors. Activated DCs also exhibit functional changes, including the downregulation of antigen uptake and secretion of chemokines and cytokines (1–6). DCs are classified as classical DCs (cDCs), which are further subdivided into classical type 1 DCs (cDC1s) and classical type 2 DCs (cDC2s), plasmacytoid DCs (pDCs), and monocyte-derived DCs (MoDCs) (7, 8). Inflammatory MoDCs are differentiated from murine bone marrow (BM) cells by treatment with granulocyte macrophage-colony stimulating factor (GM-CSF) *in vitro* (9). In contrast to the treatment with GM-CSF, BM cells treated with Flt3 ligand differentiate into cDC1s, cDC2s, and pDCs (10). GM-CSF-induced BM-derived DCs (BMDCs) are similar to *in vivo* inflammatory and TNF/iNOS-producing DCs rather than DCs in the steady state (4, 11).

GM-CSF promotes the maturation and activation of monocytes, macrophages, DCs, and granulocytes during inflammation (4, 12–14). GM-CSF also acts on the myeloid cell-dependent Th17 inflammatory response, which is mediated by TNF, IL-6, IL-23, and IL1β that are produced by macrophages and MoDCs (15–17). Besides the important roles of GM-CSF *in vivo*, it was originally identified by its ability to induce inflammatory DCs from mouse BM precursor cells *in vitro* (9, 16). The binding of GM-CSF to its receptor initiates the activation of downstream pathways, such as MAPK, PI3K/Akt, NF-κB, and STAT5. The PI3K/Akt signaling pathway regulates the GM-CSF-induced proliferation, survival, and development of DCs (17, 18).

Mst1, mammalian STE20-like kinase 1, is a multifunctional serine/threonine kinase highly expressed in immune organs, such as the thymus, spleen, and lymph nodes (19, 20). Mst1 plays important roles in cell proliferation, differentiation, apoptosis, and organ size regulation (21–24) as well as in the regulation of survival, proliferation, trafficking, and function of T cells, a type of lymphocytes in adaptive immunity (19, 20, 25–30). Furthermore, recent studies revealed that Mst1-deficient DCs promote the overproduction of IL-6, which induces Th17 differentiation in DC-specific (CD11c-Cre) conditional Mst1-knockout (KO) mice (31) and Mst1 signaling contributes to CD8^+^ DC function in mediating CD8^+^ T cell priming through the regulation of mitochondrial activity and IL-12 signaling (32). However, the roles of Mst1 in the activation and maturation of MoDCs are still poorly understood.

In this study, we aimed to elucidate the role of Mst1 in GM-CSF-induced BMDCs, which mimic *in vivo* inflammatory MoDCs more closely. We found that *Mst1*^−/−^ BMDCs displayed higher expression levels of cell surface CD40, B7, and MHC II molecules as well as increased production of inflammatory cytokines than the *Mst1*^+/+^ BMDCs. Mst1 deficiency also increased the surface expression level of B7.2 in migratory monocyte-derived cells (MCs) *in vivo*. In addition, hyperactivated *Mst1*^−/−^ BMDCs stimulated *in vitro* T cell proliferation and activation to a greater extent than *Mst1*^+/+^ BMDCs. We also found that *Mst1*^−/−^ BMDCs enhanced GM-CSF-induced Akt/c-myc pathway. Inhibition of the Akt/c-myc pathway reversed the hyperactivation of BMDCs induced by the loss of Mst1. The findings of the present study establish that Mst1 is implicated in the regulation of GM-CSF-mediated Akt/c-myc pathway in DCs, suggesting that Mst1 is one of the endogenous factors that determine the activation status of GM-CSF-stimulated inflammatory DCs. To our knowledge, this is the first observation of Akt1/c-myc-mediated functions of Mst1 in the immune system.

## Materials and Methods

### Mice

*Mst1*^−/−^ mice on the C57BL/6 background were kindly provided by Dr. Dae-Sik Lim (Korea Advanced Institute of Science and Technology, Daejeon, Korea) (33). Mice used in the experimental protocols were backcrossed more than twelve generations to C57BL/6 mice. *Mst1*^−/−^ and littermate control were maintained in a specific pathogen-free animal facility at Korea University, Seoul, Korea. These mice experiments were performed according to the guidelines of Korea University Institutional Animal Care and Use Committee (KUIACUC-2017-109 and 2019-0013).

### Chemicals and Reagents

Murine recombinant GM-CSF was purchased from ProSpec. CpG DNA (ODN 1826) was purchased from Invivogen. The following chemicals were used: Akt1 inhibitor MK-2206 (AdooQ Bioscience), myc inhibitors 10074-G5 (Cayman) and (+)-JQ1 (Sigma-Aldrich). Antibodies against the following proteins were used in western blot analyses: Mst1 (Cell Signaling, no. 3682), Mst2 (Cell Signaling, no. 3952), phospho-Akt (Cell Signaling, no. 4060), phospho-YAP (Cell Signaling, no. 13008), YAP (Cell Signaling, no. 14074), c-myc (Cell Signaling, no. 13987), Akt (Santa Cruz, sc-1618), GAPDH (Santa Cruz, sc-32233), Lamin B (Santa Cruz, sc-6217), and β-actin (Santa Cruz, sc-47778).

### Cell Numbers and Phenotyping of Primary Cells in Mouse Lymphoid Organs

Sex- and age-matched, 7- to 8-weeks old mice (female) were used in this study. Primary cells of BM, spleen, and mesenteric lymph nodes (MLN) in *Mst1*^+/+^ and *Mst1*^−/−^ littermates were used. BM cells were collected from femurs and tibias of mice. All organs were homogenized and cell suspensions were filtered through a 40-μm cell strainer for the removal of any cell clumps, and erythrocytes were removed via treatment with red blood cells lysis buffer (BioVision). For immuno-fluorescenct staining of cells, cells were resuspended in fluorescence-activated cell sorting (FACS) buffer, which consists of phosphate-buffered saline (PBS) containing 1% fetal bovine serum (FBS) and 0.05% sodium azide. We first gated on the myeloid cells based on their forward (FSC) vs. side (SSC) scatter properties, and then identify each cell type by at least two specific surface marker as follow: CD11b^+^Ly6C^+^ cells for monocytes, CD11c^+^ MHC II^+^ cells for splenic cDCs, and CD11c^+^CD103^−^CD11b^+^ cells for MCs.

### Generation of BMDCs

BMDCs were differentiated by culture of *Mst1*^+/+^ and *Mst1*^−/−^ mouse BM cells in the presence of 20 ng/ml GM-CSF for 8 days (9). Briefly, BM cells were isolated from the femur and tibia bones of *Mst1*^+/+^ and *Mst1*^−/−^ 8- to 10-weeks old mouse (female), followed by RBC lysis. The BM cells were then cultured at a concentration of 2 × 10^5^ cells per ml. The cells were cultured in RPMI containing 10% heat-inactivated FBS (Gibco), 2 mM glutamine, 1 mM sodium pyruvate, 10 mM HEPES, 100 U/ml penicillin, 100 μg/ml streptomycin (Corning), and 50 μM 2-mercaptoethanol (Sigma-Aldrich). The cells were supplemented with 20 ng/ml GM-CSF after 3, 5, and 7 days in the course of the 8-days culture or after 3, 5, 7, and 8 days in the course of the 10-days culture.

### Antibodies and Flow Cytometric Analysis

The expression of cell surface molecules on CD11c^+^ BMDCs was investigated in *Mst1*^+/+^ and *Mst1*^−/−^ BMDCs after 8 days in culture unless otherwise specified. One hundred microliters of cells were blocked with Fc blocker (anti-CD16/32 antibody, 2.4G2; BD Bioscience) for 10 min at 4°C, and then incubated with an antibody cocktail for 30 min at 4°C, and washed with FACS buffer. Fluorescent antibodies (eBioscience, BD Biosciences, and Biolegend) were used as follows: CD11c (HL3), CD11b (M1/70), B220 (RA3-6B2), Ly6c (HK1.4), MHC II (NIMR-4 and AF6-120.1), CD40 (3/23), CD80 (16-10A1), CD86 (GL1), and CD131 (JORO50) monoclonal antibodies for cell surface staining. For intracellular staining of TLR9 and CD206, *Mst1*^+/+^ and *Mst1*^−/−^ BMDCs after 8 days of culture were stained for CD11c by incubation with anti-CD11c antibody; subsequently, the cells were fixed and permeabilized using a cytofix/cytoperm kit (BD Biosciences, San Jose, CA), according to the manufacturer's instructions, and then stained with anti-TLR9 (M9.D6) or -CD206 (C068C2) antibody. The stained cells were examined by FACS Calibur (BD Biosciences, San Diego, CA), and analyzed using Cell Quest Pro software (BD Biosciences). Mst1 siRNA-mediated knockdown BMDCs were used to analyze the expression of cell surface CD40 and MHC II molecules on CD11c^+^ BMDCs after 36 h culture in the presence of GM-CSF following microporation of Mst1 siRNA.

### Semi-quantitative RT-PCR

*Mst1*^+/+^ and *Mst1*^−/−^ BMDCs after 8 days in culture were used in analysis of cytokine mRNA expression. RNAs were isolated using Ribo-EX reagent (GeneAll), as described by the manufacturer. RNA samples (1 μg) were converted to cDNA by reverse transcription using oligo (dT)18 primer and moloney murine leukemia virus (M-MLV) reverse transcriptase (Enzynomics). The PCR was performed within a range of cycles (24–37 cycles). The sequences of PCR primers used in this study are described in Table S1. In Mst1 siRNA-mediated knockdown system, BMDCs were used after 36 h culture in the presence of GM-CSF following microporation of Mst1 siRNA and media change.

### Cytokine Assay

The quantities of IL-12p40 and IL-23 in the culture supernatants were determined by a sandwich ELISA. To enhance cytokine production, BMDCs were stimulated with 0.1 μM CpG DNA. Production of the proinflammatory cytokines IL-23 and TNF-α secreted by Mst1-knockdown BMDCs was analyzed in the culture supernatants stimulated for 3 or 30 h, respectively, with 0.1 μM CpG DNA by a sandwich ELISA. Mouse IL-12p40 and TNF-α ELISA sets and anti-mouse IL-23p19 (5B2) and anti-mouse IL-12/23 p40 (C17.8) for IL-23 ELISA were purchased from eBioscience. The assays were performed according to the manufacturer's instructions.

### Quantitation of Antigen Uptake

To measure antigen uptake ability, *Mst1*^+/+^ and *Mst1*^−/−^ CD11c^+^ BMDCs after 8 days in culture were incubated with FITC-labeled dextran (Sigma-Aldrich). Briefly, 2 × 10^5^ cells were equilibrated at 4°C or 37°C for 30min and then incubated at 37°C (for a negative control, BMDCs were incubated at 4°C) in medium containing 1 mg/ml FITC-dextran for 1 h. After incubation, the cells were washed twice in PBS to remove excess dextran. The quantitative uptake of FITC-dextran by BMDCs was determined by flow cytometric analysis. We measured percentage of FITC-dextran^+^ *Mst1*^+/+^ and *Mst1*^−/−^ BMDCs following incubation after FITC-dextran treatment.

### Mixed Leukocyte Reaction (MLR)

*Mst1*^+/+^ and *Mst1*^−/−^ BMDCs (I-A^b^) after 8 days in culture were replated and cultured for 24 h in the presence of GM-CSF and used as stimulators. Allogeneic CD4^+^ T cells from spleen and lymph node of BALB/c mice (I-A^d^) were isolated by positive immunomagnetic selection using MACS with CD4 MicroBeads (Miltenyi Biotec). CD4^+^ T cells were labeled for 10 min at 37°C with 1 μM CFSE. After CFSE staining of CD4^+^ T cells, 1 × 10^5^ CD4^+^ T cells were cultured with *Mst1*^+/+^ and *Mst1*^−/−^ BMDCs at a ratio of 1:10, 20, 40, and 80 (BMDCs:CD4^+^ T cells) for 4 days in order to perform proliferation assay. The proliferation activity of CD4^+^ T cell was measured as dilution of CFSE. For detection of IL-2 production, CD4^+^ T cells were cultured with *Mst1*^+/+^ and *Mst1*^−/−^ BMDCs at a ratio of 1:10 for 3 days, and then supernatants were collected to analyze through ELISA. IL-2–expressing CD4^+^ T cells were analyzed by BD Accuri C6 Plus (BD Biosciences) at a ratio of 1:20 for 3 days after cocultured with *Mst1*^+/+^ and *Mst1*^−/−^ BMDCs.

### Small Interfering RNA (siRNA) Transfection

The siRNA-mediated interference technique was used to silence mouse Mst1 expression. The Mst1-specific sense siRNA sequence (5′-CCG UCU UUC CUU GAA UAC UUU-3′) (34) was synthesized by ST Pharm (Seoul, Korea), and a scrambled control siRNA was synthesized by Bioneer (Daejon, Korea). siRNAs were transfected into BMDCs after 8 days in culture by Neon Transfection System (Invitrogen), according to the manufacturer's instructions.

### Western Blot Analysis

*Mst1*^+/+^ and *Mst1*^−/−^ BMDCs were harvested for cell lysis after 7 days in culture. The cells were harvested and then were lysed in lysis buffer (50 mM Tris-Cl pH 8.0, 150 mM NaCl, 1% Triton X-100, 0.1% SDS, 10 mM NaF, 1 mM Na_3_VO_4_, 0.3 mM PMSF, and protease inhibitor cocktail which was from Sigma-Aldrich). Protein concentration was measured using Pierce BCA protein assay kit (Thermo Fisher Scientific), as described by the manufacturer. Equal amounts of protein from whole-cell extracts were separated on 8–10% SDS-PAGE (Bio-Rad) and transferred onto polyvinylidene difluoride (PVDF) membranes (Merck Millipore). The PVDF membrane was then incubated in blocking buffer (Tris-buffered saline containing 0.1% Tween 20 and 5% BSA) for 1 h at room temperature. Then the membranes were incubated with appropriate primary antibody overnight at 4°C with gentle shaking, followed by 1 h of incubation at room temperature with the appropriate horseradish peroxidase-conjugated secondary antibody. The blots were visualized using Amersham ECL Prime Western Blotting Detection Reagent (GE Healthcare and Life Sciences) according to the manufacturer's instructions. Western blot digital images were obtained using the Fujifilm LAS-3000 imager.

### Treatment of DCs With Inhibitors

In case of the experiments using inhibitors, the *Mst1*^−/−^ and Mst1-knockdown BMDCs were treated with each specific inhibitor. *Mst1*^+/+^ and *Mst1*^−/−^ BMDCs after 7 days of differentiation in culture were replated in the presence of GM-CSF for additional 6 h in culture. Vehicle control and MK-2206 (2 μM) were added in culture medium of *Mst1*^+/+^ and *Mst1*^−/−^ BMDCs for western blotting. For flow cytometric analysis, *Mst1*^+/+^ and *Mst1*^−/−^ BMDCs after 8 days of differentiation in culture were replated in the presence of GM-CSF and the following inhibitors: MK-2206 (2 μM), 10074-G5 (25 μM), and (+)JQ-1 (250 nM), and then annexin V^−^CD11c^+^ BMDCs were examined by BD Accuri C6 Plus after 20 h of culture. In Mst1 siRNA-mediated knockdown system, BMDCs were used after 36 h culture in the presence of GM-CSF following microporation of Mst1 siRNA and media change. Vehicle control and the indicated inhibitors were added in culture medium of scrambled control-transfected and Mst1-knockdown BMDCs during the last 6 h.

### Statistical Analyses

Statistically significant differences of all data expressed as mean ± SD were assessed by the unpaired Student *t*-test using SigmaPlot 10 software. The statistical differences in cell numbers of *Mst1*^+/+^ and *Mst1*^−/−^ mouse lymphoid organs were analyzed by Mann-Whitney U test using IBM SPSS Statistics 25 software. A *p*-value < 0.05 was considered statistically significant.

## Results

### Mst1-Deficiency Triggers a Hyperactivated Phenotype in BMDCs

A previous report showed that Mst1 is expressed abundantly in GM-CSF-induced BMDCs compared to their precursor BM cells (20). In agreement with this report, we also observed that protein levels of Mst1 in BM cells were gradually increased in a time-dependent manner when cultured with GM-CSF, which suggests that Mst1 is involved in GM-CSFR signaling (Figure S1A). Previous reports showed that Hippo pathway phosphorylates and suppresses transcriptional coactivator YAP, the component of Hippo pathway (24). We further investigated whether the protein level of YAP was altered in the BM cell differentiation into BMDCs. The protein level of YAP was inversely correlated with Mst1 expression (Figure S1A). Furthermore, the nuclear level of YAP increased in *Mst1*^−/−^ BMDCs (Figure S1B). Thus, these results suggest that Hippo pathway is activated in GM-CSF-induced BMDCs and has indispensable roles in GM-CSF-induced activation and maturation of DCs.

**Figure 1:**
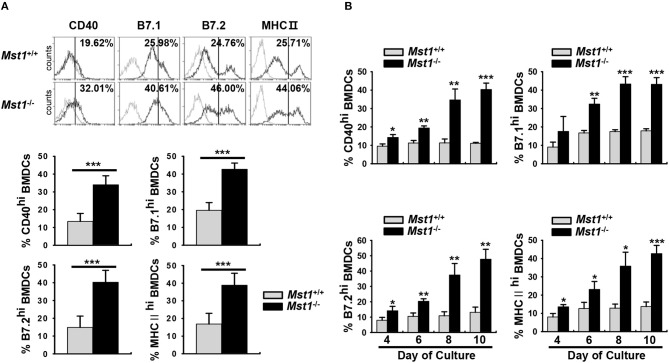
*Mst1*^−/−^ BMDCs exhibit increased levels of costimulatory and MHC II molecules. **(A,B)** BMDCs were differentiated *in vitro* from BM cells of *Mst1*^+/+^ or *Mst1*^−/−^ mice in the presence of GM-CSF. **(A)** The values in histograms indicate the percentages of CD40^+^, B7^hi^, and MHC II^hi^CD11c^+^ BMDCs. Bar graphs show the mean ± SD from at least four independent experiments. **(B)** Expression levels of CD40, B7, and MHC II on the cell surface of CD11c^+^ BMDCs were examined at the indicated time points. Bar graphs show the mean ± SD from at least three independent experiments. ^*^P < 0.05, ^**^P < 0.005, and ^***^P < 0.001 (*t*-test).

To clarify the involvement of Mst1 in the activation and maturation of DCs, we compared cell surface expression levels of costimulatory and MHC II molecules, activation/maturation-related cell surface markers, between *Mst1*^+/+^ and *Mst1*^−/−^ BMDCs. There was a greater proportion of *Mst1*^−/−^ BMDCs with high expression of the costimulatory molecules CD40, B7.1, and B7.2 (Figure 1A). Similarly, the percentage of MHC II^hi^ *Mst1*^−/−^ BMDCs was significantly higher than that of MHC II^hi^ *Mst1*^+/+^ BMDCs (Figure 1A). To further investigate the role of Mst1 in the regulation of cell surface expression of costimulatory and MHC II molecules, we compared expression levels of these cell surface molecules in *Mst1*^+/+^ and *Mst1*^−/−^ BMDCs at 4, 6, 8, and 10 days after initiation of culture. *Mst1*^−/−^ BMDCs showed an increase in the expression of the costimulatory molecules, CD40, B7.1, and B7.2, and MHC II in a time-dependent manner compared with that of *Mst1*^+/+^ BMDCs (Figure 1B). To investigate whether *Mst1*^−/−^ MoDCs display phenotypic differences *in vivo*, we compared expression levels of cell surface costimulatory molecules on MCs in the MLN of *Mst1*^+/+^ and *Mst1*^−/−^ mice. The number of migratory MCs was higher in the MLN of *Mst1*^−/−^ mice (Figure S2A). Moreover, *Mst1*^−/−^ MCs showed higher expression level of B7.2 than *Mst1*^+/+^ MCs (Figure S2B).

**Figure 2:**
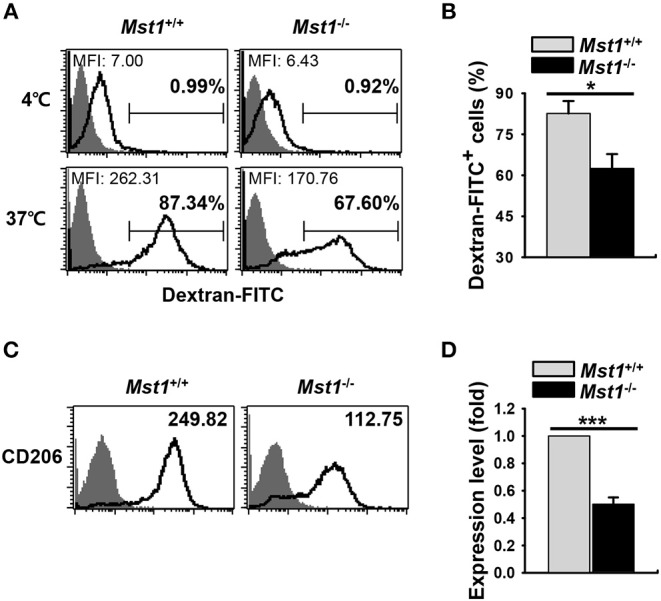
*Mst1*^−/−^ BMDCs display reduced antigen uptake. **(A,B)** The antigen uptake ability of *Mst1*^+/+^ and *Mst1*^−/−^ BMDCs was measured. *Mst1*^+/+^ and *Mst1*^−/−^ BMDCs (2 × 10^5^ cells) were incubated for 30 min at 4 or 37°C, after which the cells were further incubated for 1 h at 4 or 37°C in a medium containing 1 mg/mL of FITC-dextran. The quantitative uptake of FITC-dextran by DCs was analyzed by flow cytometry. **(A)** Numbers in the histograms represent the MFI and percentage of FITC-dextran^+^ cells. Data are representative of at least three independent experiments. **(B)** Bar graphs show the mean ± SD from three independent experiments. Filled histogram indicates unstained cells. **(C,D)** Expression level of intracellular CD206 was analyzed by flow cytometry. **(C)** Numbers in the histograms represent the MFI. Data are representative of at least three independent experiments. **(D)** Bar graphs show the fold induction in *Mst1*^−/−^ BMDCs compared to *Mst1*^+/+^ BMDCs and represent the mean ± SD from three independent experiments. Filled histogram shows isotype control. *P < 0.05 and ***P < 0.001 (*t-*test).

Decreased Ag-uptake activity and the level of mannose receptor (MR) are the hallmarks of mature DCs. Dextran is captured by pinocytosis and MR-mediated endocytosis (5, 35, 36). To compare the endocytic activity between *Mst1*^+/+^ and *Mst1*^−/−^ BMDCs, we measured the activity of MR-mediated FITC-dextran uptake. *Mst1*^−/−^ BMDCs showed decreased MFI and percentage of FITC-dextran^+^ cells in a flow cytometry analysis, suggesting a reduced antigen uptake activity of *Mst1*^−/−^ BMDCs (Figures 2A,B). In agreement with this data, the expression level of MR (CD206) was lower on *Mst1*^−/−^ BMDCs than on *Mst1*^+/+^ BMDCs (Figures 2C,D), indicating that Mst1-deficiency induced maturation of GM-CSF-derived BMDCs with the reduced endocytic activity.

**Figure 3:**
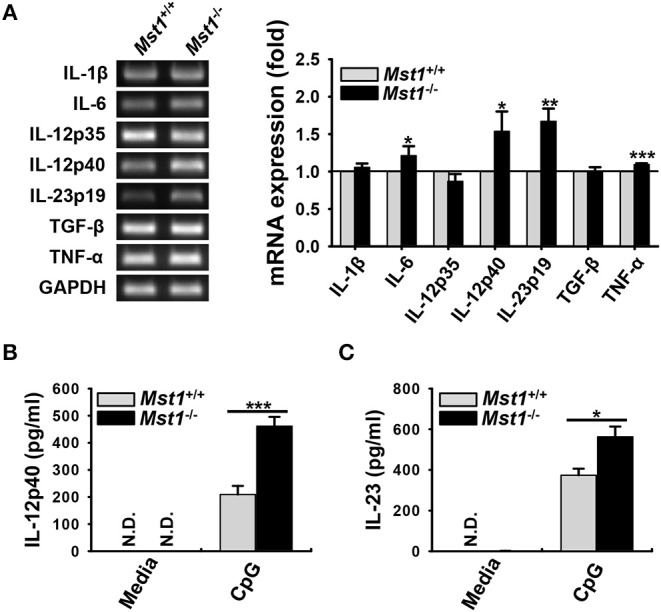
Loss of Mst1 increases production of proinflammatory cytokines in BMDCs. **(A)** mRNA expression levels of the indicated genes were analyzed by semi-quantitative RT-PCR using complementary DNA from *Mst1*^+/+^ and *Mst1*^−/−^ BMDCs generated after 8 days of culture. GAPDH served as the loading control. Data are representative of at least three independent experiments. Bar graphs show the fold induction in *Mst1*^−/−^ BMDCs compared to *Mst1*^+/+^ BMDCs and represent the mean ± SD from at least three independent experiments. **(B,C)** Levels of proinflammatory cytokines IL-12p40 **(B)** and IL-23 **(C)** secreted by *Mst1*^+/+^ and *Mst1*^−/−^ BMDCs were analyzed in the culture supernatants after stimulation for 3 h with 0.1 μM CpG DNA by ELISA. Data represent the mean ± SD from at least three independent experiments. N.D., not detected. *P < 0.05, **P < 0.005, and ***P < 0.001 (*t-*test).

DC maturation and activation are known to affect the expression of a series of inflammatory genes; as a result, they modulate subsequent immune responses (6). Therefore, we hypothesized that Mst1-deficiency leads to an overproduction of inflammatory cytokines. To explore this hypothesis, we compared mRNA expression and secretion levels of inflammatory cytokines between *Mst1*^+/+^ and *Mst1*^−/−^ BMDCs. The mRNA expression levels of IL-6, IL-12p40, IL-23p19, and TNF-α levels were higher in *Mst1*^−/−^ BMDCs than in *Mst1*^+/+^ BMDCs (Figure 3A). To confirm these increased mRNA expression levels at the protein level, we compared the production of these inflammatory cytokines by *Mst1*^+/+^ and *Mst1*^−/−^ BMDCs stimulated by CpG DNA to amplify activation. Consistent with mRNA expression levels, IL-12p40 and IL-23 production levels were notably higher in CpG-stimulated *Mst1*^−/−^ BMDCs than in *Mst1*^+/+^ BMDCs (Figures 3B,C). We excluded the possibility that the increased production of inflammatory cytokines of *Mst1*^−/−^ BMDCs stimulated through TLR may be due to an increase in TLR expression of *Mst1*^−/−^ BMDCs by determining the intracellular TLR9 expression levels of *Mst1*^+/+^ and *Mst1*^−/−^ BMDCs (Figure S3).

Taken together, Mst1-deficiency in BMDCs induced higher expression levels of cell surface molecules and proinflammatory cytokines, suggesting that Mst1 negatively regulates the activation and maturation of BMDCs.

### *Mst1*^−/−^ BMDCs Have a Greater Allostimulatory Capacity Than *Mst1*^+/+^ BMDCs

**Figure 4:**
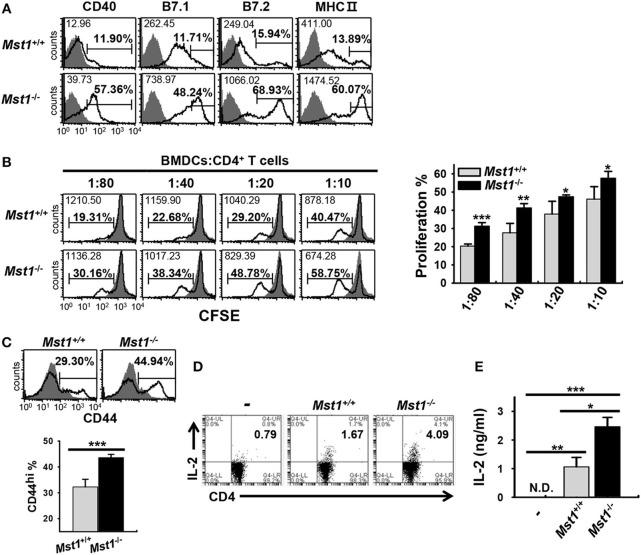
*Mst1*^−/−^ BMDCs induce stronger activation of allogeneic T cells *in vitro* than *Mst1*^+/+^ BMDCs. **(A)** Expression levels of the cell surface molecules, CD40, B7, and MHC II, on CD11c^+^ BMDCs were examined by flow cytometry after replating *Mst1*^+/+^ and *Mst1*^−/−^ BMDCs generated after 8 days of culture in the presence of GM-CSF, followed by culture for a further 24 h. The values in histograms indicate the MFI gated on CD11c^+^ cells and the percentage of CD40^+^, B7^hi^, and MHC II^hi^ CD11c^+^ BMDCs. Filled histogram shows isotype control. **(B)** Different numbers of *Mst1*^+/+^ and *Mst1*^−/−^ BMDCs (H-2K^b^) were cocultured with CD4^+^ T cells (H-2K^d^) for 4 days. Proliferation of the responding CD4^+^ T cells was evaluated by dilution of CFSE. Values in the histograms indicate the MFI and percentages of CFSE dilution. Data are representative of at least three independent experiments analyzed by flow cytometry. Bar graphs represent the mean ± SD from three independent experiments. Filled histogram shows CD4^+^ T cells cultured without stimulator BMDCs. **(C)** Allogeneic CD4^+^ T cells were cocultured for 4 days with *Mst1*^+/+^ and *Mst1*^−/−^ BMDCs at a ratio of 1:80, and the percentage of activated CD44^+^CD4^+^ T cells was measured by flow cytometry. Data are representative of at least three independent experiments. Bar-graphs show the mean ± SD from three independent experiments. Histograms are as indicated in **(A)**. **(D)** *Mst1*^+/+^ and *Mst1*^−/−^ BMDCs were cocultured for 3 days with allogeneic CD4^+^ T cells at a ratio of 1:20, and the population of IL-2-expressing CD4^+^ T cells was measured by flow cytometry. **(E)** *Mst1*^+/+^ and *Mst1*^−/−^ BMDCs were cocultured for 3 days with allogeneic CD4^+^ T cells at a ratio of 1:10. IL-2 level was determined in the culture supernatants by ELISA. Bar graphs represent the mean ± SD from three independent experiments. N.D., not detected. *P < 0.05, **P < 0.005 and ***P < 0.001 (*t-*test).

To investigate whether hyperactivated *Mst1*^−/−^ BMDCs induce a stronger activation of T cells, we compared the capacity of *Mst1*^+/+^ and *Mst1*^−/−^ BMDCs to stimulate T cells in an *in vitro* allogeneic coculture. As the activation of *Mst1*^−/−^ BMDCs was enhanced in a time-dependent manner (Figure 1B), we replated *Mst1*^+/+^ and *Mst1*^−/−^ BMDCs, generated after 8 days of culture, for a further 24 h in the presence of GM-CSF (Figure 4A) to enhance the allogeneic activity of BMDCs. As expected, *Mst1*^−/−^ BMDCs enhanced allogeneic T cell proliferation (Figure 4B). Moreover, CD4 T cells cocultured with *Mst1*^−/−^ BMDCs comprised a greater proportion of the activated CD44^hi^ T cell subset (Figure 4C). As CD4 T cells strongly produce IL-2 upon activation (37), we measured IL-2 production levels of CD4 T cells stimulated by *Mst1*^+/+^ or *Mst1*^−/−^ BMDCs. The percentage of IL-2-producing T cells and secretion level of IL-2 were higher in CD4 T cells cocultured with *Mst1*^−/−^ BMDCs than with *Mst1*^+/+^ BMDCs (Figures 4D,E). Accordingly, Mst1-deficiency in BMDCs induced the activation of allogeneic T cells to a greater extent than *Mst1*^+/+^ BMDCs *in vitro*. Collectively, these results show that Mst1 plays an important role in determining the phenotypic and functional activation degree of BMDCs.

### Mst1 Silencing in BMDCs Exhibits the Hyperactivated Phenotype Similar to That Observed in *Mst1*^−/−^ BMDCs

**Figure 5:**
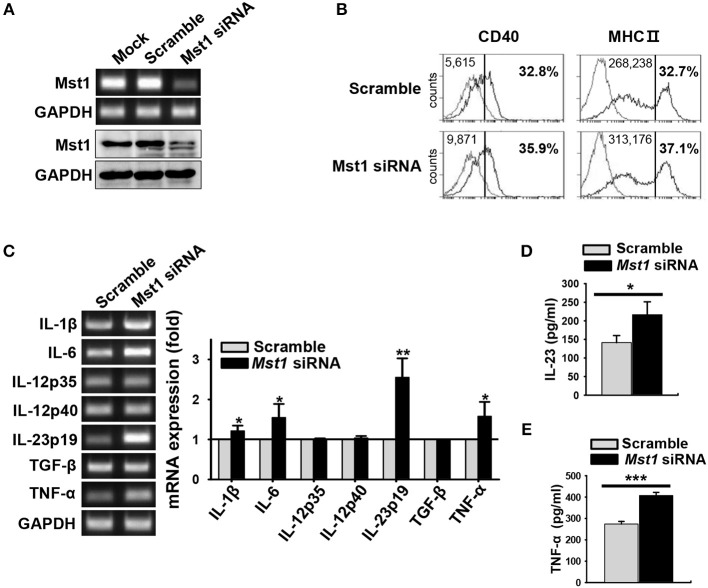
Silencing of Mst1 promotes hyperactivation of BMDCs. Mst1-specific siRNA was used for Mst1 silencing of wild-type BMDCs after 8 days of culture. **(A)** Semi-quantitative RT-PCR and western blot analysis of Mst1 expression in mock-, negative control siRNA-, and Mst1-specific siRNA-transfected BMDCs. **(B)** Expression levels of CD40 and MHC II cell surface molecules of scrambled control-transfected and Mst1-knockdown CD11c^+^ BMDCs were examined by flow cytometry. Values in the histograms indicate the MFI gated on CD11c^+^ cells and the percentages of CD40^+^ and MHC II^hi^ CD11c^+^ BMDCs. Data are representative of at least two independent experiments. Gray line shows isotype control. **(C)** mRNA expression levels of the indicated genes were analyzed by semi-quantitative RT-PCR using complementary DNA from negative control siRNA- and Mst1-specific siRNA-transfected BMDCs. GAPDH served as the loading control. Data are representative of at least three independent experiments. Bar graphs show the fold induction in Mst1-knockdown BMDCs compared to scrambled control-transfected BMDCs. Bar graphs show the mean ± SD from at least three independent experiments. **(D,E)** Levels of the proinflammatory cytokines IL-23 **(D)** and TNF-α **(E)** secreted by Mst1-knockdown BMDCs were analyzed in the culture supernatants stimulated for 3 h (for IL-23) or 30 h (for TNF-α) with 0.1 μM CpG DNA by ELISA. Data shown represent the mean ± SD from at least three independent experiments. *P < 0.05, **P < 0.005, and ***P < 0.001 (*t-*test).

Next, we confirmed phenotype of the hyperactivated *Mst1*^−/−^ BMDCs in an Mst1-specific siRNA-mediated knockdown system. The efficiency of Mst1 silencing was validated by semi-quantitative RT-PCR (Figure 5A, top) and western blot analysis (Figure 5A, bottom). Silencing of the Mst1 gene in BMDCs increased the surface expression levels of CD40 and MHC II molecules (Figure 5B). The mRNA expression levels of IL-1β, IL-6, IL-23p19, and TNF-α were increased in Mst1-knockdown BMDCs (Figure 5C). In agreement with these data, secretion of these proinflammatory cytokines was enhanced in Mst1-knockdown BMDCs, with IL-23 and TNF-α levels significantly elevated (Figures 5D,E). Accordingly, silencing of Mst1 promoted BMDC hyperactivation, similar to the hyperactivation observed in *Mst1*^−/−^ BMDCs. To perform these Mst1-knockdown experiments, we transfected siRNA targeting Mst1 into BMDCs differentiated after 8 days of culture, which are different from *Mst1*^−/−^ BMDCs in which Mst1 is absent in cells before the differentiation of BM cells into BMDCs. Differentiation rates and cell numbers of BM cells into BMDCs between *Mst1*^+/+^ and *Mst1*^−/−^ cells were similarly obtained (Figures S4A,B), which means that *Mst1*^−/−^ BMDCs normally differentiate from BM cells. Furthermore, both *Mst1*^+/+^ and *Mst1*^−/−^ BMDCs induced by GM-CSF had no difference in the percentages of CD11c^+^B220^+^ pDC population (Figure S4C). It convinced us to exclude the possibility that BMDC hyperactivation is due to an effect of Mst1-deficiency on cell development *in vitro*. These results are consistent with the idea that Mst1-deficiency in differentiated BMDCs, and not in precursors, gives rise to their hyperactivation. Taken together, these data suggest that endogenous Mst1 in differentiated BMDCs suppresses their hyperactivation.

### Hyperactivated Phenotype Induced by Loss of Mst1 in BMDCs Is Not Due to a Change in GM-CSFR Expression

**Figure 6:**
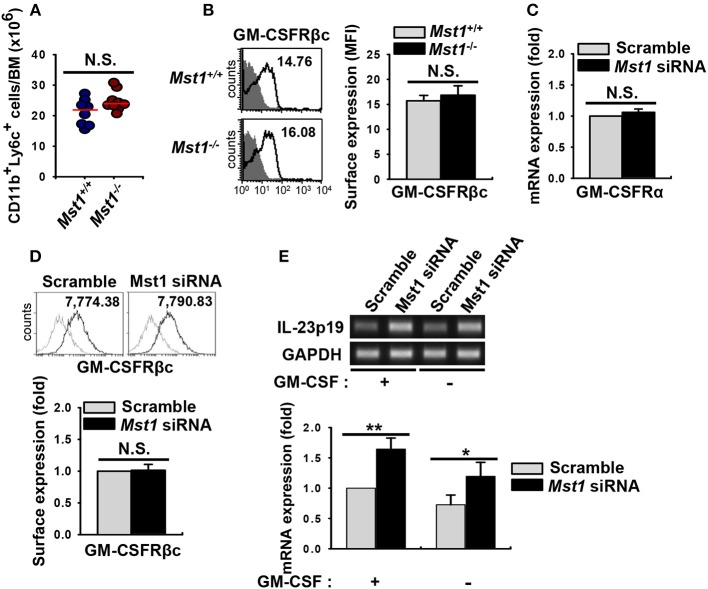
Loss of Mst1 shows similar levels of GM-CSFR expression. **(A)** Total cell numbers of monocyte (CD11b^+^Ly6C^+^ in the myeloid gate) population in the BM of *Mst1*^+/+^ (blue circles) and *Mst1*^−/−^ (red circles) mice. Data are pooled from eight independent experiments and each dot represents the data obtained from one mouse (*n* = 8–9 mice); horizontal lines indicate the median. Statistical significance was determined by Mann-Whitney *U* test. N.S., not significant. **(B)** Expression of GM-CSFRβc on monocytes was determined in the BM of *Mst1*^+/+^ and *Mst1*^−/−^ mice by flow cytometry. The values in histograms indicate the MFI gated on CD11b^+^Ly6C^+^ monocytes. Histogram data are representative of at least four independent experiments. Bar graphs show the mean ± SD from at least four independent experiments. Filled histogram shows isotype control. **(C)** Semi-quantitative RT-PCR analysis of GM-CSFRα expression in negative control siRNA- and Mst1-specific siRNA-transfected BMDCs. Bar graphs show the fold induction in Mst1-knockdown BMDCs compared to scrambled control siRNA-transfected BMDCs. Bar graphs show the mean ± SD from at least three independent experiments. **(D)** Expression levels of cell surface GM-CSFRβc of scrambled control-transfected and Mst1-knockdown CD11c^+^ BMDCs were examined by flow cytometry. Values in the histograms indicate the MFI gated on CD11c^+^ cells. Data are representative of at least three independent experiments. Gray line shows isotype control. Bar graphs show the fold induction in Mst1-knockdown compared to control-transfected CD11c^+^ BMDCs and represent the mean ± SD from three independent experiments. **(E)** mRNA expression level of IL-23p19 was analyzed by semi-quantitative RT-PCR using complementary DNA from negative control siRNA- and Mst1-specific siRNA-transfected BMDCs in the absence or presence of GM-CSF (10 ng/ml). Bar graphs show the fold induction of the indicated conditions compared to scrambled control-transfected BMDCs in the presence of GM-CSF. *P < 0.05 and **P < 0.005 (*t-*test).

GM-CSF is involved in the inflammatory phenotype of DCs (4, 12, 13). The α subunit of GM-CSF receptor (GM-CSFRα) recruits GM-CSFRβc (38), which results in the initiation of GM-CSF signal transduction and activation of downstream pathways followed by regulation of the development, survival, and activation of GM-CSF-induced DCs (18). We first compared the absolute cell numbers of monocytes, the main precursor of BMDCs, in BM of *Mst1*^+/+^ and *Mst1*^−/−^ BMDCs. Cell numbers of monocytes were comparable in BM from *Mst1*^−/−^ and *Mst1*^+/+^ mice (Figure 6A). Next, we checked the expression level of cell surface GM-CSFRβc, which transmit GM-CSF signaling, to check whether the hyperactivation of *Mst1*^−/−^ BMDCs was due to hyperresponsiveness of monocytes to GM-CSF. The expression of cell surface GM-CSFRβc was slightly increased in *Mst1*^−/−^ monocytes but not significantly (Figure 6B). Mst1-knockdown BMDCs showed comparable mRNA expression level of GM-CSFRα (Figure 6C) and the cell surface expression level of GM-CSFRβc (Figure 6D). As expected, the mRNA expression level of IL-23p19 increased in Mst1-knockdown BMDCs, regardless of the presence of GM-CSF (Figure 6E). These data suggest that responsiveness to GM-CSF does not play a role in the hyperactivation of BMDCs induced by the loss of Mst1.

### Mst1-Deficiency Causes Hyperactivation of BMDCs Through Enhanced Akt1/c-myc Signaling

**Figure 7:**
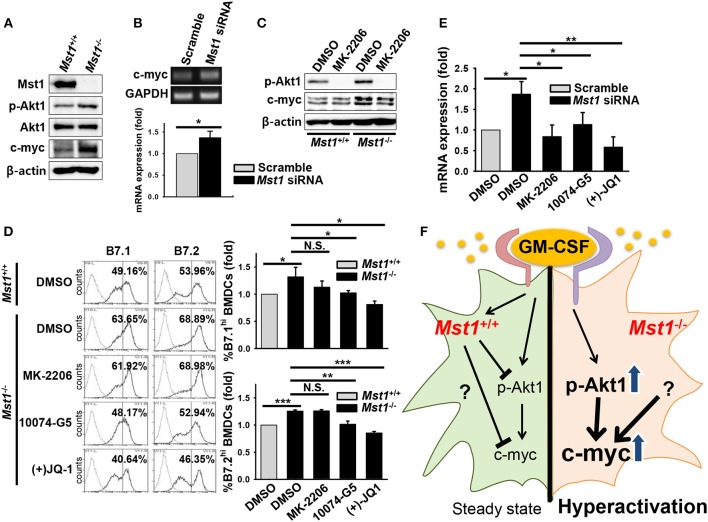
Mst1-deficiency causes hyperactivation of BMDCs through enhanced Akt1/c-myc axis. **(A)** Protein levels of p-Akt1 (S473), total Akt1, and c-myc were investigated in whole cell lysates of *Mst1*^+/+^ and *Mst1*^−/−^ BMDCs after 7 days of differentiation in culture. β-actin served as the loading control. **(B)** Gene expression of c-myc was analyzed by semi-quantitative RT-PCR from scrambled control-transfected and Mst1-knockdown BMDCs treated with MK-2206 (2 μM). GAPDH served as the loading control. Data are representative of at least three independent experiments. Bar graphs show the fold induction in Mst1-knockdown BMDCs compared to scrambled control-transfected BMDCs and represent the mean ± SD from at least three independent experiments. **(C)** *Mst1*^+/+^ and *Mst1*^−/−^ BMDCs after 7 days of differentiation in culture were replated in the presence of GM-CSF for additional 6 h in culture. DMSO and MK-2206 (2 μM) were added in culture medium of *Mst1*^+/+^ and *Mst1*^−/−^ BMDCs. Protein levels of p-Akt1 (S473), total Akt1, and c-myc were determined in whole cell lysates of the indicated cells. **(D)** *Mst1*^+/+^ and *Mst1*^−/−^ BMDCs after 8 days of differentiation in culture were replated in the presence of GM-CSF and the following inhibitors: MK-2206 (2 μM), 10074-G5 (25 μM), and (+)JQ-1 (250 nM). Expression levels of the cell surface B7.1 and B7.2 molecules on annexin V^−^CD11c^+^ BMDCs were examined by flow cytometry after 20 h of culture. DMSO served as the vehicle control. Data are representative of at least two independent experiments. Gray line shows isotype control. **(E)** mRNA expression level of IL-23p19 was analyzed by semi-quantitative RT-PCR using complementary DNA from scrambled control-transfected and Mst1-knockdown BMDCs treated with the indicated inhibitors. Bar graphs show the fold induction in Mst1-knockdown BMDCs treated with DMSO and the indicated inhibitors compared to scrambled control-transfected BMDCs treated with DMSO. Bar graphs represent the mean ± SD from at least three independent experiments. *P < 0.05, **P < 0.005, and ***P < 0.001 (*t*-test). **(F)** Proposed model for Mst1-mediated regulation of the activation status in GM-CSF-stimulated DCs. Mst1 negatively regulates the hyperactivation of GM-CSF-induced inflammatory DCs. Mst1-deficiency enhances GM-CSF-activated Akt1 (phosphorylation of S473), which induces de novo synthesis of c-myc. Increased expression level of c-myc, which might mediate strong metabolism, is responsible for the hyperactivation of GM-CSF-stimulated DCs. Collectively, Mst1 is involved in the hyperactivation of GM-CSF-stimulated DCs via downregulation of the Akt1/c-myc axis.

Previous reports showed that Mst1 antagonizes Akt1 activation in various cell types (39–41), including regulatory T cells in which FoxO1/3 proteins, directly and indirectly regulated by Mst1, are involved in their development (29). We hypothesized that Mst1-deficiency triggers the hyperactivation of GM-CSF-induced BMDCs by regulating Akt1 activity. To test this hypothesis, we investigated Akt1 activity in Mst1-deficient BMDCs after 7 days in culture. Consistent with our hypothesis, the phosphorylation of Akt1 increased in *Mst1*^−/−^ BMDCs compared to that in *Mst1*^+/+^ BMDCs (Figure 7A). Akt1-mediated regulation of c-myc expression plays a crucial role in the determination of an inflammatory phenotype of GM-CSF-induced macrophages (42), and contributes DC development, regulating survival and maturation (43). Therefore, we compared the protein level of c-myc in *Mst1*^+/+^ and *Mst1*^−/−^ BMDCs. After 7 days in culture, the protein level of c-myc increased in *Mst1*^−/−^ BMDCs (Figure 7A). We confirmed the gene induction of c-myc in Mst1-knockdown BMDCs. The mRNA expression level of c-myc also increased in Mst1-knockdown BMDCs compared to that in cells transfected with a scrambled control (Figure 7B). To investigate whether Akt1 is a potent inducer of c-myc, *Mst1*^+/+^ and *Mst1*^−/−^ BMDCs after 7 days in culture were further cultured in the presence of GM-CSF and MK-2206. The treatment of BMDCs with MK-2206 decreased the phosphorylation of Akt1, and partially reversed the protein level of c-myc in *Mst1*^−/−^ BMDCs (Figure 7C). Together, these results indicate that Akt1 is involved in the de novo synthesis of c-myc in Mst1-deficient DCs.

Next, to determine whether the Akt1/c-myc axis is responsible for the hyperactivation of *Mst1*^−/−^ BMDCs, we compared expression levels of the costimulatory molecules, B7.1 and B7.2, on the cell surface of *Mst1*^−/−^ BMDCs treated with a vehicle control or the indicated inhibitors. As Akt1 and c-myc are involved in the survival of DCs, we excluded the dead cells in this comparison. *Mst1*^−/−^ BMDCs treated with inhibitors of c-myc reversed the increase in expression levels of the costimulatory molecules in *Mst1*^−/−^ BMDCs although MK-2206 failed to decrease expression levels of the costimulatory molecules in *Mst1*^−/−^ BMDCs (Figure 7D). To further investigate whether the Akt1/c-myc axis mediates the hyperactivation of BMDCs induced by the loss of Mst1, the mRNA expression level of IL-23p19 was compared in Mst1-knockdown BMDCs treated with the vehicle control or the indicated inhibitors. The mRNA expression level of IL-23p19 in Mst1-knockdown BMDCs treated with inhibitors of Akt1 and c-myc was downregulated compared to that in Mst1-knockdown BMDCs treated with the vehicle control (Figure 7E). Therefore, these data suggest that the enhanced Akt1/c-myc signaling is responsible for the hyperactivation of *Mst1*^−/−^ BMDCs. Thus, Mst1 negatively regulates the Akt1/c-myc axis, which determines the inflammatory phenotype of GM-CSF-induced DCs.

## Discussion

Given that DCs are used in vaccination, connecting innate and antigen-specific responses, understanding how the maturation and activation of DCs are regulated is important (14, 44, 45). Mst1 is a multifunctional serine/threonine kinase involved in cell proliferation, differentiation, apoptosis, and organ size regulation (21–24). Several recent studies have revealed crucial roles for Mst1 in the immune system; specifically, it regulates the survival, proliferation, trafficking, and function of T cells (19, 20, 25–30). Although previous studies have revealed that Mst1 is involved in the induction of reactive oxygen species to clear bacterial infection in macrophages (46) and in the production of IL-6 (31) and IL-12 (32) from DCs, the roles of Mst1 in the activation and maturation of MoDCs are still largely unknown. In the present study, we aimed to clarify the intrinsic role of Mst1 in the determination of the activation status of GM-CSF-induced inflammatory DCs. We found that *Mst1*^−/−^ BMDCs exhibited an increased expression of costimulatory and MHC II molecules and production of several inflammatory cytokines *in vitro*; moreover, the results of Mst1 knockdown in BMDCs are consistent with the idea that Mst1 suppresses the overexpression of several inflammatory cytokines and cell surface molecules in fully differentiated BMDCs. In conclusion, our results suggest that Mst1 negatively regulates the phenotypical and functional activation of GM-CSF-induced DCs.

DCs have functional properties depending on their maturation status. Their distinctive intrinsic properties lead to maturation of different subsets (2, 11, 47, 48). Furthermore, the previous study showed that Mst1 is the negative regulator of proliferation in naïve T cells (19) and regulates development and function of regulatory T cells (29). To investigate whether the hyperactivation of *Mst1*^−/−^ GM-CSF-derived DCs is due to a differential development, we compared cell numbers and percentages of the CD11c^+^CD11b^+^ population in *Mst1*^+/+^ and *Mst1*^−/−^ BMDCs. Comparable percentages and cell numbers of the CD11c^+^CD11b^+^ population at an earlier culture time were observed (Figures S4A,B). The previous study showed that GM-CSF suppresses the differentiation of pDCs (49). Consistent with the previous study, we observed that both *Mst1*^+/+^ and *Mst1*^−/−^ BMDCs induced by GM-CSF had no apparent percentage of CD11c^+^B220^+^ pDC population (Figure S4C). Thus, these data show that *Mst1*^−/−^ BMDCs from mouse BM cells normally differentiate into CD11c^+^CD11b^+^ inflammatory DCs, which suggests that Mst1 has a redundant role in the *in vitro* differentiation of BMDCs by GM-CSF. Taken together, these data demonstrate that BMDC hyperactivation is not due to an effect of Mst1-deficiency on cell development *in vitro*.

In the present study, we did not observe any abnormal death of Mst1-KO mice as a result of a spontaneous autoimmune response *in vivo*, which seems inconsistent with the *in vitro* hyperactivation of *Mst1*^−/−^ GM-CSF-induced DCs. Previous reports have reported a systemic T cell lymphopenia due to defects in homing and survival in Mst1-KO mice (20, 25, 50), and Mst1-mutated patients with impaired T cell survival that resulted in primary T cell immunodeficiency (26). However, a recent study has revealed that DC-specific (CD11c-Cre) conditional Mst1-KO mice exhibit overproduction of IL-6 by DCs, inducing Th17 differentiation and autoimmune response *in vivo* (31). We speculate that the inconsistency between the normal phenotype of Mst1-KO mice and the hyperactivation of *Mst1*^−/−^ BMDCs *in vitro* may have several explanations. First, Mst1-KO mice have severe T cell lymphopenia in peripheral lymphoid organs (data not shown), which is consistent with the previous reports (19, 20). Second, consistent with a previous report (32), we did not observe any critical differences in numbers and phenotypic changes of DCs in the spleen of Mst1-KO mice (Figure S5). Finally, the critical roles of GM-CSF in inflammation rather than steady state *in vivo* might explain the absence of spontaneous autoimmune responses in Mst1-KO mice.

These findings shed light on our understanding of the physiological role of Mst1 in the regulation of activation status of GM-CSF-induced DCs. Mst1 dampens the hyperactivation of BMDCs by regulating the Akt1/c-myc axis rather than GM-CSFR expression. A previous report showed that Mst1 antagonizes Akt1 activation in regulatory T cells in which FoxO1/3 proteins that are directly and indirectly regulated by Mst1 act on their development (29). Thus, we hypothesized that Mst1-deficiency triggers an enhanced activity of Akt1, which results in the hyperactivation of BMDCs. As expected, phosphorylation of Akt1 was increased in *Mst1*^−/−^ BMDCs (Figure 7A), and the blockade of Akt1 activity reversed the hyperactivation of Mst1-knockdown BMDCs (Figure 7E). Akt1 is also known for controlling the cellular metabolism. Activation-induced T cell metabolic reprogramming (51) and glycolytic metabolism in TLR-activated DCs (52) have been suggested, with clear evidence of the involvement of cellular metabolism in immune cell function. The PI3K/Akt1-induced transcription factor, c-myc, is a regulator of cellular metabolism, especially glycolysis, and thereby of the activation of macrophages (42). Moreover, c-myc, the downstream effector of mTORC1, is involved in the development of DCs (43). Thus, we tested whether c-myc is involved in the hyperactivation of *Mst1*^−/−^ BMDCs. Consistent with our hypothesis, we demonstrated elevated protein (Figure 7A) and mRNA (Figure 7B) levels of c-myc in Mst1-deficient BMDCs. Inhibitors of c-myc reversed the hyperactivation of Mst1-KO (Figure 7D) and knockdown (Figure 7E) in BMDCs. Although previous studies showed that PI3K/Akt signaling regulates GM-CSF-induced proliferation, survival, and development of DCs (18), we observed a normal development and yield of CD11b^+^CD11c^+^ *Mst1*^−/−^ BMDCs in an *in vitro* culture (Figure S4), which means that the Mst1/Akt1/c-myc pathway has a redundant role in the proliferation and differentiation of BM precursor cells into BMDCs, whereas it is required to maintain a moderate maturation phenotype of GM-CSF-induced DCs. Collectively, Mst1-deficiency triggers the hyperactivation of BMDCs through the overactivation of GM-CSF-induced Akt1/c-myc signaling pathway.

We observed that the treatment of Mst1-deficient BMDCs with Akt1 inhibitor partially decreased the protein level of c-myc (Figure 7C) and also failed to reverse expression levels of the costimulatory molecules (Figure 7D), which means that the blockade of Akt1 activity was not sufficient to suppress c-myc-mediated hyperactivation in GM-CSF-stimulated DCs. The recovery of increased costimulatory B7 expression levels in *Mst1*^−/−^ BMDCs might be required for complete inhibition of c-myc expression, even though the modest reduction of c-myc level was sufficient to reverse the mRNA expression level of IL-23p19, a proinflammatory cytokine. Thus, although we have elucidated one crucial mechanism underlying inhibition of hyperactivation of GM-CSF-stimulated DCs, we expect that unknown other mediators might also exist.

In summary, we have demonstrated that Mst1 dampens the hyperactivation of DCs via downregulation of the Akt1/c-myc axis in response to GM-CSF, suggesting that Mst1 in mouse inflammatory DCs correlates with GM-CSF-driven disease state. The Mst1/Akt1/c-myc pathway in the regulation of DC activation will give a new insight into understanding of the way how Mst1 regulates appropriate immune responses. These findings shed light on how the maturation and activation of DCs are regulated by a novel endogenous serine/threonine kinase factor. Furthermore, since GM-CSF-induced DCs are a key player in inflammation and autoimmunity (17), Mst1 can be a new and considerable therapeutic target in the treatment of GM-CSF-derived inflammatory diseases, such as multiple sclerosis, rheumatoid arthritis, and inflammatory bowel disease (17, 53).

## Data Availability

The datasets generated for this study are available on request to the corresponding author.

## Ethics Statement

The experimental protocols adopted in this study were approved by the Institutional Animal Care and Use Committee of Korea University.

## Author Contributions

K-MC designed and performed all the experiments. K-MC, MK, and TK analyzed and interpreted the experimental results. H-JJ maintained the mice used in this study. E-JC played a role in discussing the results and provided crucial Mst1-related reagents. K-MC and TK collaborated on the manuscript writing. TK supervised the study and corrected the manuscript.

### Conflict of Interest Statement

The authors declare that the research was conducted in the absence of any commercial or financial relationships that could be construed as a potential conflict of interest.
