# Supplementary material for: Mst1-Deficiency Induces Hyperactivation of Monocyte-Derived Dendritic Cells via Akt1/c-myc Pathway
Source: Front Immunol. 2019 Sep 11;10:2142. doi: 10.3389/fimmu.2019.02142 (PMC6749027; doi:10.3389/fimmu.2019.02142)
Supplement: Supplementary file 1 [file Data_Sheet_1.ZIP › KMCho.Mst1.DC.FigS1-5.Revised.20190815.pdf]

Supplementary Figure S1

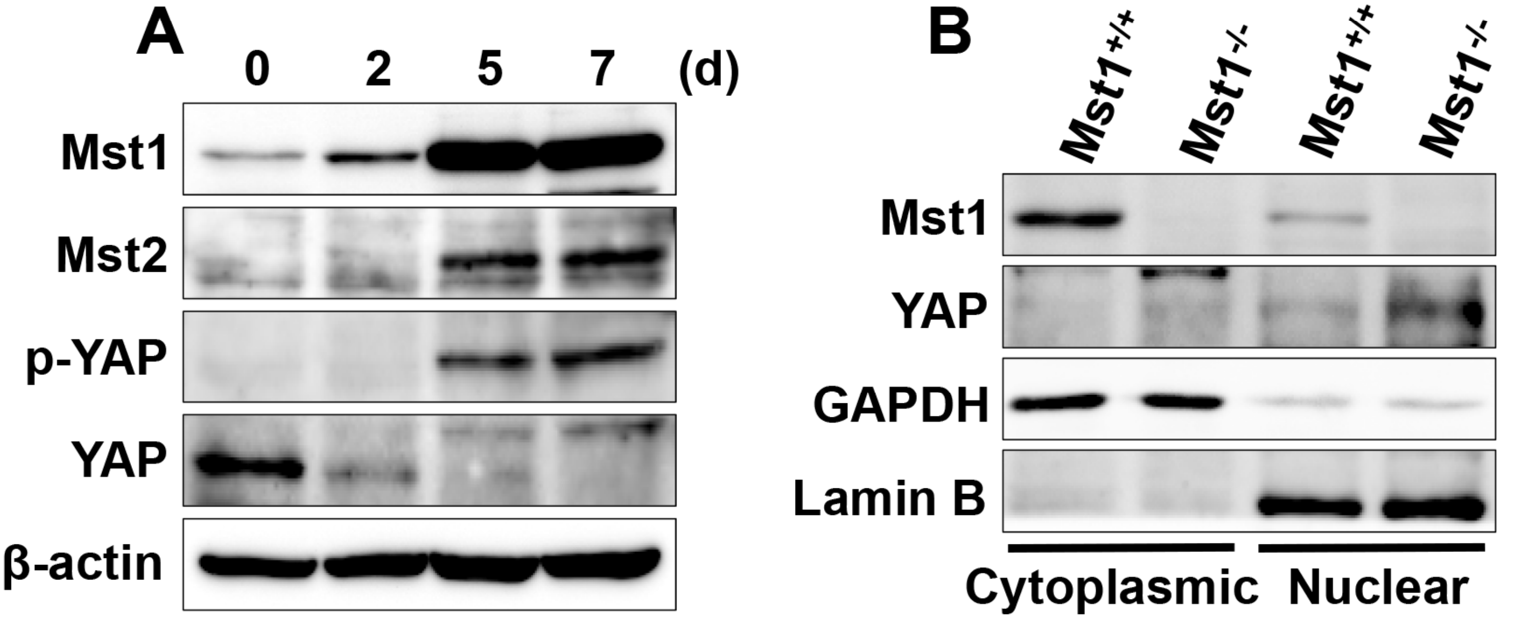

Figure S1. Hippo pathway is activated in GM-CSF-induced BMDCs. **(A and B)** BM cells were cultured in the presence of 20 ng/ml GM-CSF to differentiate into BMDCs. **(A)** Protein levels of Mst1, Mst2, p-YAP (S127), and total YAP were determined in whole cell lysates after 3, 5, and 7 days of differentiation in culture. β-actin served as the loading control. **(B)** Protein levels of Mst1 and YAP were investigated in cytoplasmic and nuclear extracts of *Mst1*$^{+/+}$ and *Mst1*$^{-/-}$ BMDCs after 8 days of differentiation in culture. GAPDH (cytoplasmic) and Lamin B (nuclear) served as the loading control.

Supplementary Figure S2

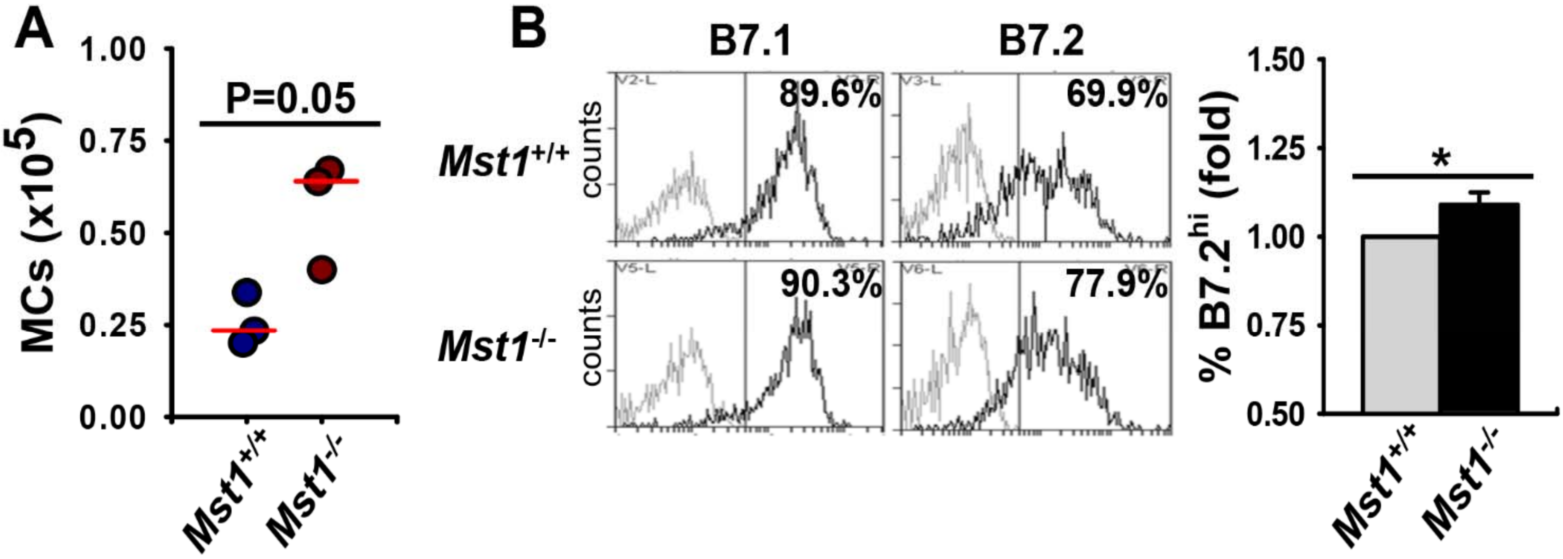

Figure S2. *Mst1*$^{-/-}$ mouse increases the surface expression level of B7.2 on MCs in the MLN. **(A)** Total cell number of MC (CD11c^{+}CD103^{-}CD11b^{+}) population in the MLN of *Mst1*$^{+/+}$ (blue circles) and *Mst1*$^{-/-}$ (red circles) mice. Data are pooled from three independent experiments and each dot represents the data obtained from one mouse (n = 3 mice); horizontal lines indicate the median. Statistical significance was determined by Mann-Whitney U test. **(B)** The expression levels of cell surface B7.1 and B7.2 molecules on MCs were determined in the MLN of *Mst1*$^{+/+}$ and *Mst1*$^{-/-}$ mice by flow cytometry. The values in histograms indicate the percentages gated on MCs. Histogram data are representative of at least three independent experiments. Bar graphs indicate fold induction of *Mst1*$^{-/-}$ cells compared to *Mst1*$^{+/+}$ cells. Data represent the mean ± SD from at least three independent experiments. Gray line shows isotype control.

Supplementary Figure S3

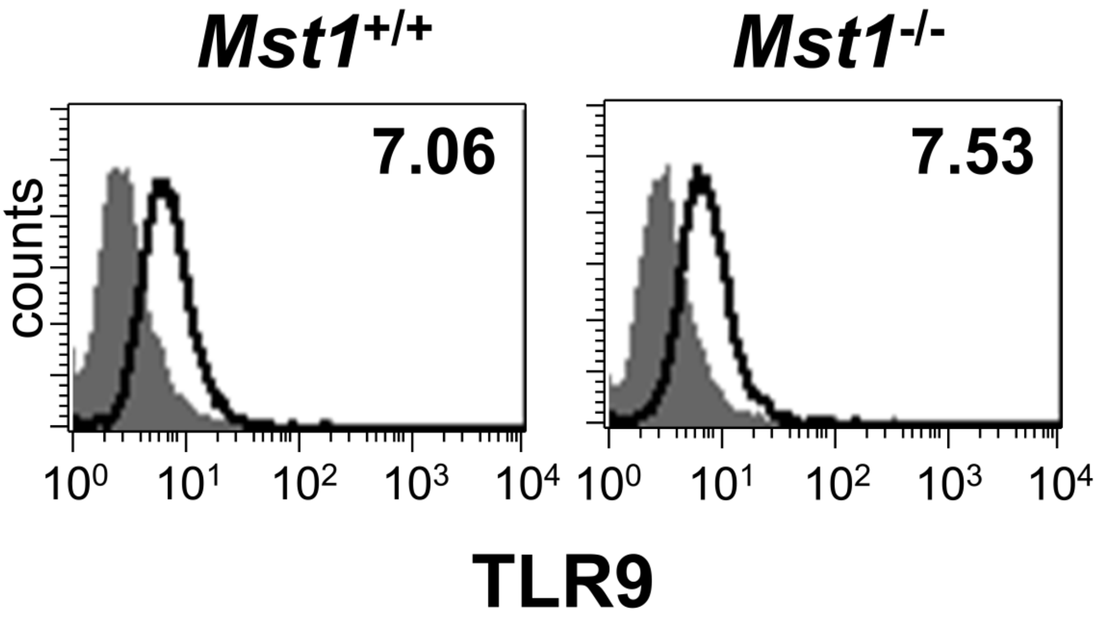

Figure S3. *Mst1*$^{-/-}$ BMDCs exhibit similar levels of intracellular TLR9 expression compared to *Mst*$^{+/+}$ BMDCs. Expression level of intracellular TLR9 molecule was determined on *Mst1*$^{+/+}$ and *Mst1*$^{-/-}$BMDCs after 8 days in culture by flow cytometry. The values in histograms indicate the MFI gated on CD11c^{+} BMDCs. Histogram data are representative of two independent experiments. Filled histogram shows isotype control.

Supplementary Figure S4

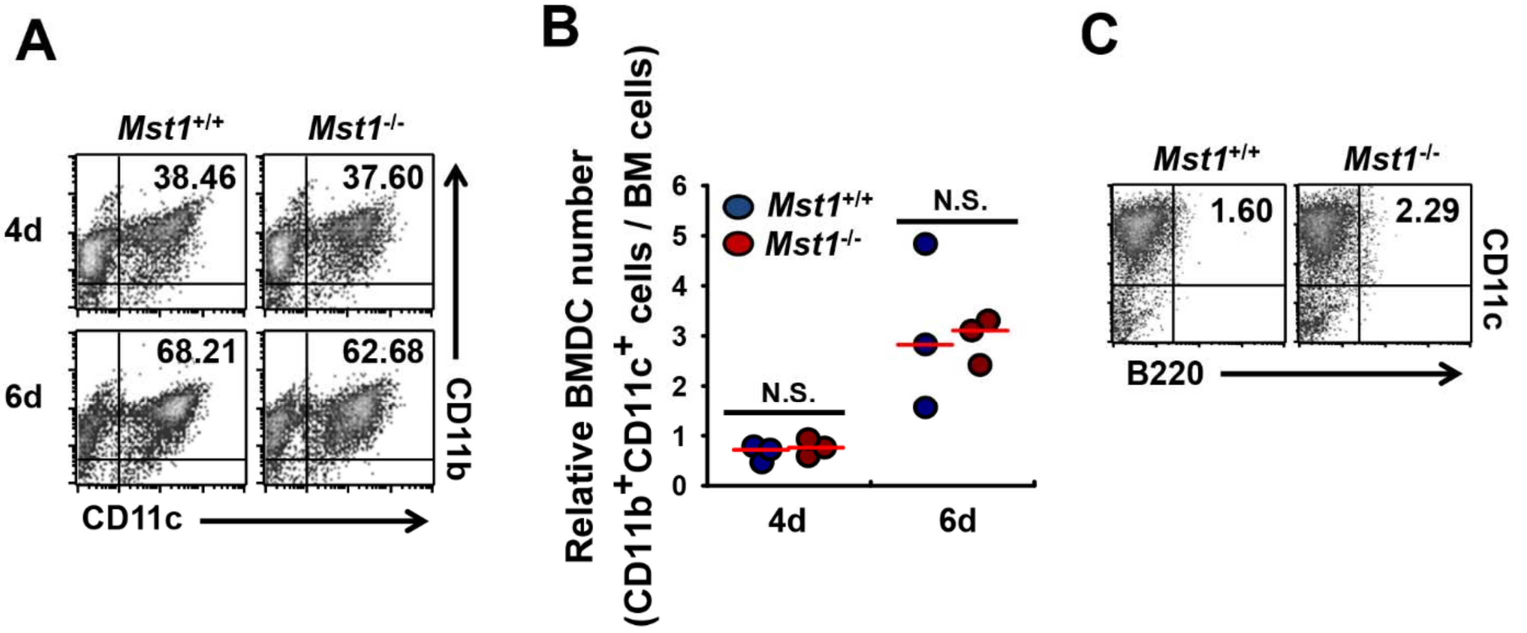

Figure S4. *Mst1*$^{-/-}$ BMDCs normally differentiate into CD11c^{+}CD11b^{+} myeloid cDCs. **(A and B)** BMDCs were differentiated by culture of *Mst1*$^{+/+}$ and *Mst1*$^{-/-}$ mouse BM cells for 4 and 6 days. The percentages of CD11c^{+}CD11b^{+} cells were measured by flow cytometry **(A)** and total cell numbers of CD11c^{+}CD11b^{+} population in *Mst1*$^{+/+}$ (blue circles) and *Mst1*$^{-/-}$ (red circles) BMDCs were represented **(B)**. Data are pooled from three independent experiments and each dot represents the data obtained from one independent experiment; horizontal lines indicate the median. Mann-Whitney U test was carried out to determine the statistical differences in cell numbers of *Mst1*$^{+/+}$ and *Mst1*$^{-/-}$ BMDCs. **(C)** The percentages of CD11c^{+}B220^{+} cells were measured by flow cytometry.

Supplementary Figure S5

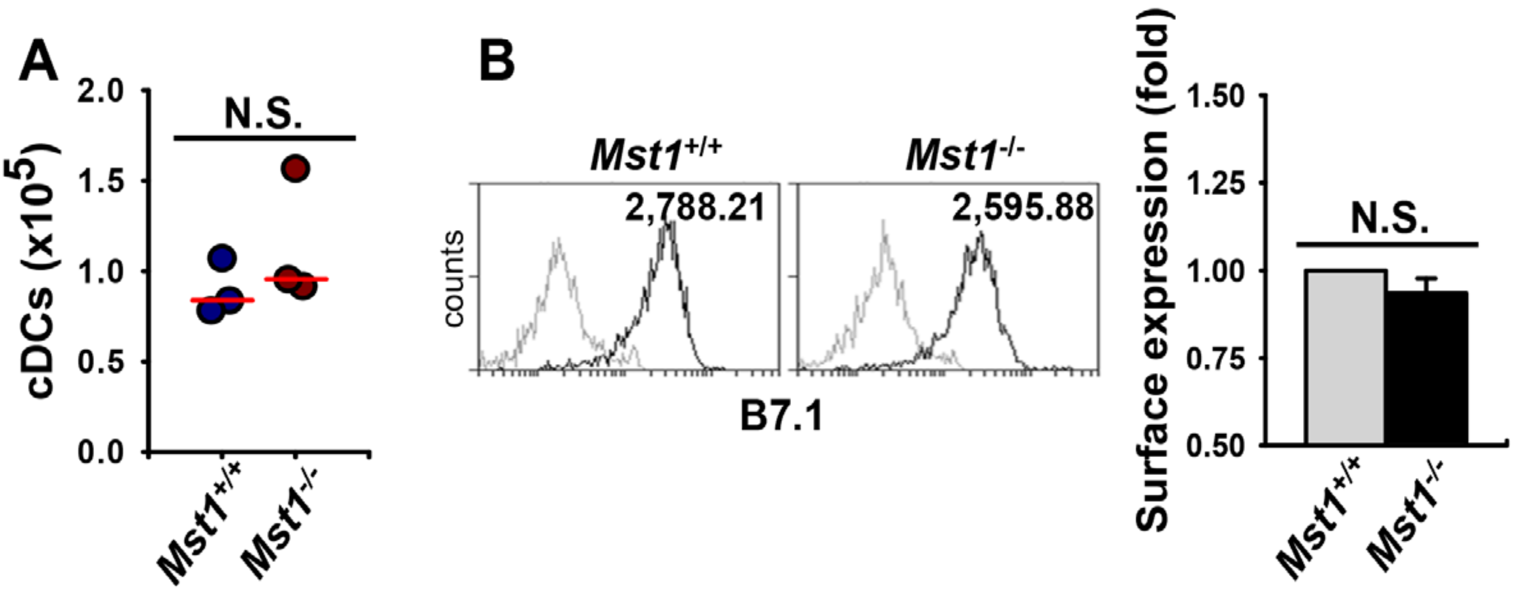

Figure S5. *Mst1*$^{-/-}$ mouse shows the comparable cell number and surface expression level of B7.1 in splenic cDCs. **(A)** Total cell number of cDC (CD11c$^+$MHC II$^+$ in the myeloid gate) population in the spleen of *Mst1*$^{+/+}$ (blue circles) and *Mst1*$^{-/-}$ (red circles) mice. Data are pooled from three independent experiments and each dot represents the data obtained from one mouse (n = 3 mice); horizontal lines indicate the median. Statistical significance was determined by Mann-Whitney U test. N.S., not significant. **(B)** The cell surface expression of B7.1 on cDCs was determined in the spleen of *Mst1*$^{+/+}$ and *Mst1*$^{-/-}$ mice by flow cytometry. The values in histograms indicate the MFI gated on cDCs. Histogram data are representative of at least three independent experiments. Bar graphs indicate fold induction of *Mst1*$^{-/-}$ cells compared to *Mst1*$^{+/+}$ cells. Data represent the mean ± SD from at least three independent experiments. Gray line shows isotype control.
