# Supplementary material for: Mst1-Deficiency Induces Hyperactivation of Monocyte-Derived Dendritic Cells via Akt1/c-myc Pathway
Source: Front Immunol. 2019 Sep 11;10:2142. doi: 10.3389/fimmu.2019.02142 (PMC6749027; doi:10.3389/fimmu.2019.02142)
Supplement: Supplementary file 1 [file Data_Sheet_1.ZIP › KMCho.Mst1.DC.TableS1.20190527.pdf]

Supplementary Table S1

Gene	Primer sequence (5'-3')
Mst1	FW, GACAGCCCTCACGTAGTCAA<br>RV, AGGAGCCATCCAAAACGGG
IL-1β	FW, TGCCACCTTTTGACAGTGATG<br>RV, GCTTGTGCTCTGCTTGTGAG
IL-6	FW, TGAACA ACGATGATGCACTT<br>RV, CGTAGAGAACAACATAAGTC
IL-12p35	FW, CCAAGGTCAGCGTTCCAACA<br>RV, CCAAGGCACAGGGTCATCATCA
IL-12p40	FW, TGTGGAATGGCGTCTCTGTC<br>RV, CCTTTGCATTGGACTTCGGTAG
IL-23p19	FW, GACTCAAGGACAACAGCCAGT<br>RV, CAAACAAAACAAGAACAGCACAAGTC
TGF-β	FW, TATAGCAACAATTCCTGGCGT<br>RV, TCCTAA AGTCAATGTACAGCT
TNF-α	FW, GGCAGGTCTACTTTGGAGTC<br>RV, ACA TTCGAGGCTCCAGTGAA
Csf2ra	FW, CTCTCGAGGCTGAGGACACG<br>RV, GAGCAAACTATTACGGTGGGGAT
Myc	FW, CCTTCTCTCCTTCCTCGGACT<br>RV, CCGCCTCTTGTCGTTTTCCT
GAPDH	FW, ACATCAAGAAGGTGGTGAAG<br>RV, ATTCAAGAGAGTAGGGAGGG

Table S1. Primers for semi-quantitative RT-PCR
